# Impairment of translation in neurons as a putative causative factor for autism

**DOI:** 10.1186/1745-6150-9-16

**Published:** 2014-07-10

**Authors:** Eugenia Poliakov, Eugene V Koonin, Igor B Rogozin

**Affiliations:** 1 Laboratory of Retinal Cell & Molecular Biology, National Eye Institute, National Institutes of Health, Bethesda, MD, USA; 2 National Center for Biotechnology Information, National Library of Medicine, National Institutes of Health, Bethesda, MD, USA

**Keywords:** Synonymous mutations, Single nucleotide polymorphism, Codon usage, Splicing enhancer, Splicing silencer, mRNA secondary structure, Transcription factor binding, Neurotoxin

## Abstract

**Background:**

A dramatic increase in the prevalence of autism and Autistic Spectrum Disorders (ASD) has been observed over the last two decades in USA, Europe and Asia. Given the accumulating data on the possible role of translation in the etiology of ASD, we analyzed potential effects of rare synonymous substitutions associated with ASD on mRNA stability, splicing enhancers and silencers, and codon usage.

**Presentation of the hypothesis:**

We hypothesize that subtle impairment of translation, resulting in dosage imbalance of neuron-specific proteins, contributes to the etiology of ASD synergistically with environmental neurotoxins.

**Testing the hypothesis:**

A statistically significant shift from optimal to suboptimal codons caused by rare synonymous substitutions associated with ASD was detected whereas no effect on other analyzed characteristics of transcripts was identified. This result suggests that the impact of rare codons on the translation of genes involved in neuron development, even if slight in magnitude, could contribute to the pathogenesis of ASD in the presence of an aggressive chemical background. This hypothesis could be tested by further analysis of ASD-associated mutations, direct biochemical characterization of their effects, and assessment of *in vivo* effects on animal models.

**Implications of the hypothesis:**

It seems likely that the synergistic action of environmental hazards with genetic variations that in themselves have limited or no deleterious effects but are potentiated by the environmental factors is a general principle that underlies the alarming increase in the ASD prevalence.

**Reviewers:**

This article was reviewed by Andrey Rzhetsky, Neil R. Smalheiser, and Shamil R. Sunyaev.

## Background

Autism prevalence in USA increased from 1 in 2,000 in 1970 to 1 in 68 in 2010 [1]. The CDC estimate for Autistic Spectrum Disorders (ASD) among school aged children (8 years) has increased by 78% during 2002–2008 (from 1 in 156 to 1 in 88) [2]. A major increase in the prevalence of autism was observed over the last two decades also in Asia and Europe [3].

The causes of such an apparent explosion of ASD are a subject of intense debate. Several recent studies examined the rate of *de novo* mutations in autistic children and their possible importance in the etiology of ASD [4-7]. Comparison of ASD-affected individuals and their parents and/or unaffected siblings revealed modest but significant excess of *de novo* mutations in ASD, particularly in genes that are specifically expressed in the brain [4]. These findings imply a substantial genetic component in the etiology of ASD but fail to shed light on the recent dramatic increase in the prevalence of autistic disorders. In general, the rate of mutations linked to classic Mendelian and complex diseases does not seem to have significantly increased within the relevant time span [8-10]. For example, the rate of *de novo* mutations associated with Huntington’s disease has been estimated at steady 8–10% of all cases over the last decade [11,12]. Congruent with this observation, no obvious increase in the incidence of common diseases with a major genetic component, e.g. schizophrenia, haemophilia A or cystic fibrosis, has been detected over the last 50 years [13-15]. Thus, germline mutations alone hardly can explain the recent explosion of autism [16]. Another potential factor, change in diagnostic criteria, such as inclusion of milder cases, might explain at best approximately 25% of the observed increase in ASD incidence [17-20].

By exclusion, it has been proposed that environmental factors including chemicals and microbes could be the primary culprits in the ASD surge [3,18,21-23]. Recent twin studies have shown that susceptibility to ASD has moderate heritability (38%) and a substantial shared twin environmental component (58%) [24]. Early twin studies had estimated the heritability of autism to be as high as 90 percent, due to much lower estimates of concordance - both members of a twin pair having the disorder - in fraternal twins [25-28]. New studies found the concordance among fraternal twins to be four to five fold higher [24,29]. Such a dramatic difference could be explained by smaller sample size in previous twin studies and/or a genuine drop in heritability caused by additional environmental factors that became involved in the etiology of autism in the last twenty years [3].

The changes in environmental factors that occurred during the last two decades have been substantial and numerous. The primary suspects so far are mercury [23], agricultural pesticides [30], air pollutants and solvents [31,32], proximity of maternal residence at the time of delivery to freeways [33], toxins present in plastic and treatment of mothers with selective serotonin reuptake inhibitors, thalidomide, valproic acid and misoprostol [34-36]. However, none of these environmental factors has been firmly established as a contributor to the ASD surge [37].

## Presentation of the hypothesis

Recently, it has been shown that mutations in translational repressors FMRP, TSC1/2 and PTEN [38,39] as well as *de novo* disruptions of FRMP-regulated genes [7] are associated with ASD, suggesting that defects in translation repression could be a causative factor. Given the accumulating data on the possible role of translation in the etiology of ASD, we analyzed rare synonymous substitutions linked to disorders of the ASD spectrum (delineated from rare gene variations, see below for more details) because these mutations could directly reflect selection at the levels of transcription and/or translation [40]. We assessed potential impact of ASD-associated synonymous substitutions on mRNA stability, splicing enhancers/silencers and codon usage (see below for more details). The results indicate a statistically significant shift in codon usage associated with ASD: a substantial majority of rare synonymous variants change an optimal codon to a sub-optimal one. We hypothesize that subtle impairment of translation resulting from the codon usage shift contributes to the ASD surge through interaction with increasingly used neurotoxic compounds.

## Testing the hypothesis

### Features of synonymous variants associated with ASD

Rare synonymous variations in human genes associated with ASD (the AV set) were selected from the AutDB database [41] and from a set of rare synonymous variations unique to ASD patients that was compiled by Kelleher and co-workers from the AGRE database [42] (Table 1). These variations were polarized under the assumption that a rare variant implicated in ASD is a derived state whereas the alternative common allele is the ancestral state [41,42]. Altogether, 87 synonymous AVs in 19 genes were used for further analysis (Additional file 1). We also reconstructed a set of fixed synonymous substitutions for the same set of human genes (Table 1) using multiple alignments of orthologs from humans, chimp and gorilla. For each gene, we compared triplets of species including two sister species (human and chimp) and an outgroup (gorilla). A substitution was assumed to have occurred in the human lineage if the nucleotides in the chimp lineage and in the gorilla outgroup were identical but differed from the nucleotide in the human lineage. The use of parsimony is justified in this case because the distances between the three species of great apes are short [43]. We additionally analyzed common synonymous single nucleotide polymorphisms (SNPs) from the dbSNP database for the same group of genes (Table 1), a set of substitutions that is expected to be strongly enriched for neutral or nearly neutral variants [44]. These common SNPs were polarized using chimp/gorilla as an outgroup. Furthermore, we examined 99 recently identified *de novo* synonymous mutations in ASD patients [6,7]. The vast majority of these mutations are unlikely to cause any functional consequences [6,7,45] and accordingly these mutations were used as an additional control.

**Table 1:** The 19 genes with ASD-associated rare synonymous variations

**Gene**	**Source database**	**GenBank protein GI/brief description**	**# rare synonymous variants (AVs)**	**# reconstructed synonymous mutations**	**# common synonymous SNPs**
AUTS2	AutDB	17225457/autism-related protein 1	1	2	2
CADPS2	AutDB	148839294/calcium-dependent secretion activator 2	2	5	3
FOXP2	AutDB	298566291/forkhead box protein P2	1	4	2
FMR1	AutDB	fragile X mental retardation 1	1	4	3
GRM1	AGRE	166999098/metabotropic glutamate receptor 1	4	8	5
GRM5	AGRE	4504143/metabotropic glutamate receptor 5	7	3	5
HRAS	AGRE	34222246/GTPase HRas	1	0	1
MAP2K1	AGRE	5579478/dual specificity mitogen-activated protein kinase 1	1	0	1
MAP2K2	AGRE	13489054/dual specificity mitogen-activated protein kinase 2	3	4	4
MECP2	AutDB	1708973/Methyl-CpG-binding protein 2	9	2	1
NLGN3	AutDB	262359971/neuroligin-3	1	0	1
NRXN1	AutDB	154813843/neuronal cell surface protein NRXN1-α	5	10	2
PIK3CA	AGRE	54792082/phosphatidylinositol 4,5-bisphosphate 3-kinase catalytic subunit	4	2	3
RBFOX1	AutDB	22538409/RNA binding protein fox-1 homolog 1	3	6	1
SHANK2	AutDB	254763402/SH3 and multiple ankyrin repeat domains protein 2	8	6	3
SHANK3	AutDB, AGRE	380748963/SH3 and multiple ankyrin repeat domains protein 3	27	4	9
TSC1	AGRE	4507693/tuberous sclerosis gene TSC1	3	2	2
TSC2	AGRE	116256352/tuberous sclerosis 2 protein	5	11	8
UBE3A	AGRE	19718764/ubiquitin-protein ligase E3A	1	3	5

We compared the properties of the AV to those of the other mutations (Table 2). The AV are enriched in C:G > T:A transitions (Table 2) but the difference from the set of reconstructed mutations, common SNPs and *de novo* mutations was not significant [46] (Table 2). The frequency of mutations in the CpG context is higher in the AV set (48%) compared to the set of reconstructed mutations (25%), and this difference was statistically significant after correction for multiple comparisons (Table 2). The excess of mutations in CpG sites might reflect subtle differences in the methylation pattern between the AVs and the reconstructed mutation group although problems with the parsimony reconstruction, such as excess of multiple parallel mutations in CpG codons in chimp and gorilla, cannot be ruled out. No significant differences in the CpG site mutations were observed between the AV set and *de novo* synonymous mutations or common SNPs (Table 2). Regardless of its biological relevance, the observed excess of mutations in the CpG dinucleotides and obvious biases in substitution patterns (Table 2) should not be ignored in any analysis of AVs. These biases were taken into account in the analysis of codon usage, splicing enhancers/silencers and free energy as described below.

**Table 2:** Features of the analyzed synonymous variations/mutations

	**1. Rare variations from AutDB/AGRE (AVs)**	**2. Reconstructed mutations**	**3. Common SNPs**	**4. *De novo *** **mutations**
C:G > T:A	64	44	34	66
C:G > A:T	7	6	8	6
C:G > G:C	4	3	6	6
T:A > C:G	10	20	9	16
T:A > G:C	1	3	3	3
T:A > A:T	1	3	1	2
P_MCχ2_ = 0.390, P_ctcχ2_ = 0.378, P_Zd_ = 0.464				
Mutations in CpG’s (total #mutations)	42 (out of 87)	20 (out of 79)	24 (out of 61)	42 (out of 99)
P_Fisher_ for two columns (C’s)		C1 vs. C2	C1 vs. C3	C1 vs. C4
		0.002	0.316	0.462

A Monte Carlo modification of the Pearson *χ*2 test (MCχ2) of spectra homogeneity, CTC *χ*2 and Zd measures were used for comparison of mutation frequencies as implemented in the COLLAPSE program [46]. Two-tailed Fisher exact test was used to compare frequencies of substitutions in CpG sites.

For the analysis of the changes in codon usage, we used multiple sources of codon frequencies in brain-specific genes (denoted here as F). The codon frequencies were inferred from two sets of brain-specific genes that were delineated using expressed sequence tags [47] and microarray data [48,49]. Codon frequencies were also calculated for the set of 19 AV genes (Table 1). The shift in codon usage (*S*_*H*_) between common allele variants (C-CV) and polarized rare synonymous variants (C-AV) was calculated using the equation:

(1) $$S_{H}=\sum_{i=1}^{N}\frac{\left({F^{i}}_{C‒\mathrm{CV}}{{‒F}^{i}}_{C‒\mathrm{AV}}\right)}{{F^{i}}_{C‒\mathrm{CV}}}$$

where *F*^*i*^_*C-CV*_ and *F*^*i*^_*C-AV*_ are frequencies of codons *C-CV* and *C-AV*, and *N* is the number of variations (mutations) in the analyzed dataset. Under the assumption that more frequent codons are translated more efficiently [40,48,50-54], positive *S*_*H*_ values imply that translation of mutated codons is less efficient.

A Monte Carlo procedure was used to estimate the significance of the differences in *S*_*H*_. Random synonymous mutations were simulated taking into account the frequencies of substitutions for each nucleotide and the context of the mutating sites in the case of CpG dinucleotides (distinguishing mutations in the first and second CpG positions). A distribution of S_H_ _r values was calculated for 10,000 groups of random mutations. Each of the resulting random mutation spectra contained the same number of mutations as the observed AV set with the same distribution of mutation types (Table 2) over randomly chosen synonymous sites for each gene. The distribution of S_H_ _r was used to calculate the probability P(S_H_ ≤ S_H__r). This probability is equal to the fraction of random spectra in which S_H__r is the same as or greater than the S_H_. Similarly, P(S_H_ ≤ S_H__r) values for the sets of reconstructed synonymous mutations, common SNPs and *de novo* mutations were calculated.

The same Monte Carlo approach was applied to analyze the potential impact of AVs on mRNA free energy and on splicing enhancers. The mRNA sequences of 19 genes (Table 1) were computationally “folded” and the predicted minimum free energy of the secondary structure was calculated using an algorithm that employs nearest neighbor parameters to evaluate free energy [55,56]. Similar to codon usage, the change in free energy divided by the value of the free energy of common allele variants was used as S_H_ = Σ (FE_C_CM_ – FE_C_AV_)/FE_C_CM_ (FE_C_CM_ is the minimum free energy of a common variant, FE_C_AV_ is the free energy of AV). Lists of splicing enhancers/silencers were from [57,58]. The S_H_ was defined as the number of changes of splicing enhancers/silencers (gains or losses of splicing enhancers/silencers as the result of variations).

### No effect of AVs on splicing enhancers/silencers, mRNA secondary structure and exonic transcription factor binding

Selection at synonymous sites in mammals is a long-standing problem. It has been shown that some synonymous sites in mammalian genes are subject to selective pressure, possibly because of constraints acting on regulatory elements (such as splicing enhancers) located within exons [51,58-64]. Exonic splicing enhancers (ESEs) and silencers (ESSs) appear to be present in most, if not all, mammalian exons [59-61]. In the present analysis, no connection between ESEs/SSEs and the AVs was detected: P(S_H_ ≤ S_H__r) = 0.213 for the ESE/SSE set [58] and P(S_H_ ≤ S_H__r) = 0.713 for the RESCUE-ESE set [57].

It also has been proposed that purifying selection on synonymous sites had to do with changes in mRNA stability [40,51,65-67]. Thus, we compared the predicted mRNA structures for the polarized rare synonymous variations in the AV set and the common variants. No significant stabilization or destabilization of the mRNA structure in the AVs was detected [P(S_H_ ≤ S_H__r) = 0.189]. Thus, stability of mRNA structure or ESEs/ESSs does not seem to be significantly affected by the AVs.

Recently, it has been shown that exonic transcription factor binding directs codon choice through the dual function of many codons that additionally contribute to transcription factor binding site [68]. The dual function codons are concentrated primarily in the 5′-terminal regions of protein-coding sequences. Therefore we addressed the possibility that AVs affected transcription factor binding. The distribution of AVs across the coding regions of the 19 genes was nearly uniform (Additional file 2) which is inconsistent with a significant effect of AVs on transcription factor binding sites.

### The AVs result in less frequent codons

**Figure 1:**
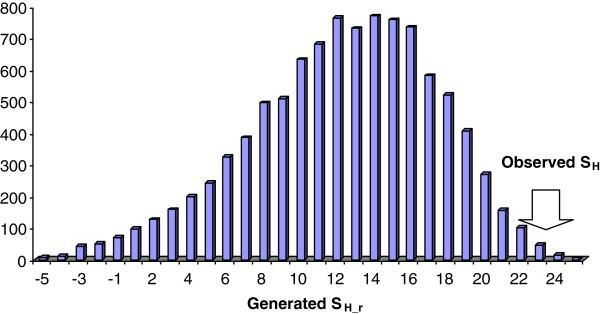
**Distribution of the mean codon usage shift (S**_**H**_**_r) for 10,000 groups of generated mutations.** Codon usage shift (S_H_) for observed synonymous mutations: 22.7, P(S_H_ ≤ S_H__r) = 0.007. Monte Carlo stimulation was used to estimate the significance of changes in codon usage, taking into account DNA context properties of mutations (see text for details). Codon frequencies for the brain-specific genes were estimated by Semon and co-workers [47].

In some metazoan species (e.g. Drosophila and nematode), synonymous codon usage appears to evolve under selective pressure to optimize the efficiency of translation of highly expressed genes (the translational selection model) [69]. The impact of translational selection on synonymous codon usage in mammals is uncertain, and codon usage is often considered to be effectively neutral [47,51,69,70]. However, recent analyses indicate that selection on translation efficiency in mammals could be measurable as suggested by the detected correlations between codon adaptation index and several variables related to the functional importance of proteins including gene essentiality, expression level and evolutionary rate [52,53]. Most of the AVs cause replacement of more frequent codons by less frequent codons (68 out of 87 AVs). However, this trend might result from mutational biases [47], especially given that many variations occurred in CpG dinucleotides (Table 2). We used Monte Carlo stimulations to estimate the significance of changes in codon usage, taking into account DNA context properties of mutations (see above). The distribution of shifts in codon usage for random mutations (S_H__r) is shown in Figure 1. The S_H_ value for the AVs is 22.7, and only a small fraction of S_H__r (0.007) showed equal or greater values (Figure 1). Thus, the observed shift in codon usage associated with AVs is unlikely to be due to chance [P(S_H_ ≤ S_H__r) = 0.007]. We complemented this analysis using codon frequencies in the set of 19 AV-containing genes (Table 1) and the microarray-based set of brain-specific genes [48], with nearly identical results [P(S_H_ ≤ S_H__r) = 0.006 for the 19 gene set and P(S_H_ ≤ S_H__r) = 0.005 for the alternative brain-specific gene set].

A comparable codon usage shift was not observed for *de novo* mutations [P(S_H_ ≤ S_H__r) = 0.171]. These *de novo* mutations are an important control because the vast majority of them are not related to ASD and rather reflect properties of spontaneous mutations in humans [6,7,45]. No significant codon usage shift was detected for the set of reconstructed synonymous mutations [P(S_H_ ≤ S_H__r) = 0.636] and the set of common SNPs [P(S_H_ ≤ S_H__r) = 0.291] either. Thus, the shift in codon usage toward rare codons is not a general property of spontaneous mutations in the analyzed set of 19 ASD-associated genes (Table 1).

One obvious limitation of this analysis is the small numbers of variations/mutations (Table 2). To assess the impact of the dataset size, we applied the jackknife procedure: X mutations were randomly removed from the AV set and P(S_H_ ≤ S_H__r) was estimated for this smaller subset of AVs. This procedure was repeated 1000 times for each X (X = 10, 20, 26, 30, 40; X = 26 corresponds to the smallest set of common synonymous SNPs, Table 2). The results of the jackknife analysis are shown in the Table 3. For X = 10, 20, 26, 30, the vast majority of P(S_H_ ≤ S_H__r) values were smaller than 0.05 (significant codon usage shift); only for X = 40, the results were notably less reliable even though for all X values, the mean P value was below 0.05 (Table 3). Thus, the relatively small size of the AV dataset and other datasets analyzed here does not preclude reliable conclusions on the significance of the codon usage shift. It should be noted that careful selection and validation of the genes associated with ASD and the AVs themselves provided by the authors of the initial datasets [41,42] compensated for the small size of the AV set.

**Table 3:** Jackknife analysis of the rare synonymous variations in genes associated with ASD (the AV set)

**Number of removed AVs**	**Mean P(S**_**H **_**≤ S**_**H**_**_r)**	**Standard error**	**Fraction of cases with P(S**_**H **_**≤ S**_**H**_**_r) ≤ 0.05**
10	0.0031	0.0004	0.988
20	0.0233	0.0007	0.939
26 (corresponds to the common SNP dataset)	0.0279	0.002	0.886
30	0.0323	0.002	0.839
40	0.0451	0.003	0.718

Codon frequencies for the brain-specific genes were estimated by Semon and co-workers [47].

## Implications of the hypothesis

**Figure 2:**
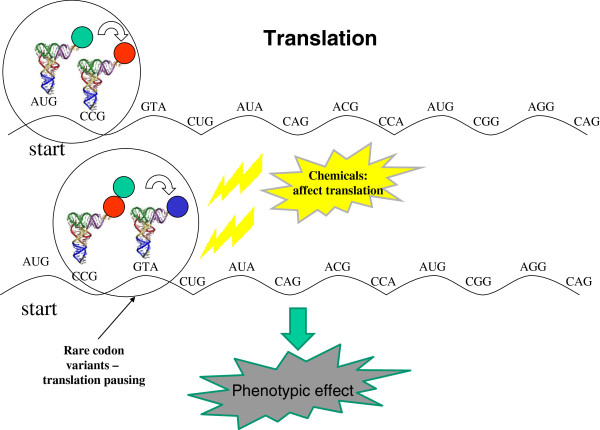
**Schematic representation of the interplay between the rare codons in human genes associated with ASD and increased exposure to an unidentified neurotoxin(s).** These rare variations in genes involved in neuron development are hypothesized to contribute to the pathogenesis of ASD in the presence of aggressive chemical background whereas in the absence of such background these rare variations are unlikely to cause noticeable phenotypic effects.

We show here that rare synonymous variations in 19 genes associated with ASD cause a significant shift of codon usage towards rare codons and therefore might reduce the efficiency of translation. An example of such rare synonymous variation was found in the MDR1 gene; this rare synonymous variation results in the protein product with altered drug and inhibitor interactions [71,72]. We hypothesize that the impact of rare codons on the translation of genes involved in neuron development, even if slight in magnitude, could contribute to the pathogenesis of ASD in the presence of an aggressive chemical background (Figure 2). In several single-gene disorders with a high prevalence of autism, the mutated gene products are negative regulators of protein synthesis [38]. For example, the product of the FMR1 gene, the fragile X mental retardation protein (FMRP), binds to specific mRNAs and represses their translation. The FMRP protein is estimated to interact with more than 400 mRNAs [73]. These findings are consistent with the observation that *de novo* disruptions of genes regulated by the FRMP translation repressor are prevalent in children with ASD [7]. Kelleher and Bear proposed that the “autistic neuron” is a result of perturbed translation and the defects in translation repression might represent one possible etiological factor of autism [7,38,74,75].

Rare codons can substantially alter the dosage of proteins through different mechanisms, in particular ribosome pausing and “drop off” from the mRNA, often followed by decay of the mRNA and the partly completed protein product [76,77]. Dosage imbalance of proteins associated with neuron development is known to be important in many psychiatric disorders [78-80]. Currently, there seem to be no direct indications that dosage imbalance of neuron-specific proteins is involved in ASD. However, indirect evidence includes multiple reports on apparent involvement of molecular chaperones, in particular the heat shock protein 70 kDa family (Hsp70) that facilitates cotranslational folding of large, slowly translated and aggregation-prone proteins [81-83]. Prolonged episodes of fever are known to cause induction of heat shock protein response along with a two-three fold increased risk of infantile ASD [84-86]. Neurotoxicity also leads to heat shock protein response including Hsp70 induction [87]. Elevation of the Hsp70 level in rat brain homogenates has been detected in response to the treatment with the neurotoxin propionic acid [88] that has been used to develop a rat model of ASD [89]. Micromolar concentrations of another neurotoxin, sodium azide, in human cell cultures induce a cytoplasm to nucleus translocation and overall upregulation of Hsp70 [90]. *In utero* exposure to valproic acid, a mood stabilizer, is associated with increased risk of autism in humans and autistic-like behaviors in rodent models [91,92]. Valproic acid treatment induces functional Hsp70 in cortical rat neuron cell cultures [93]. Conceivably, the induction of Hsp70 by factors associated with ASD might contribute to protein imbalance that is likely to play a role in the etiology of this disease.

Under the present hypothesis (Figure 2), the ASD surge is, at least partially, caused by the interplay between human genetic background, in particular that associated with perturbed translation, and increased exposure to an unidentified neurotoxin(s). Many growing environmental hazards could be at fault [34-37]. It seems likely, however, that the synergistic action of such hazards with mutations that in themselves have limited or no deleterious effects but are potentiated by the environmental factors is a general principle that underlies the alarming increase in the ASD prevalence.

Some aspects of this hypothesis can be tested via further epidemiological studies, direct biochemical experiments aimed at the elucidation of the effects of synonymous substitutions, in particular in relation to stress and chaperone activity, and the *in vivo* effects of these mutations in animal models.

## Reviewers’ comments

### Reviewer #1: Andrey Rzhetsky, University of Chicago, United States of America

Every hypothesis should have an opportunity to be presented for future testing with data; I would support an opportunity to voice this particular suggestion. I enjoyed reading the introduction to the paper--it covers well the diversity of proposed hypothetical genetic and environmental etiologies for ASD.

However, the particular hypothesis proposed by the authors does not really explain the mechanism of disease. In its essence, this is a limited genetic explanation (limited to a subset of protein-coding nucleotide substitutions). It does not account for increase of the apparent ASD rate (except the factors reviewed in the introduction, but the hypothesis is disconnected from these factors). Essentially, the authors suggest that mildly deleterious synonymous substitutions in protein-coding genes related to neurodevelopmental functions change the efficiency of translation process. The idea is that closer-to-the-optimum (corresponding to the maximum-concentration tRNA isoacceptor) codons are replaced with less optimal ones, slowing translation. It is unclear what the authors think about more serious defects in the same genes (non-synonymous, nonsense, missense, etc.)? Would they result in the same phenotype or be lethal or neutral?

Authors’ response: The presented hypothesis is not actually disconnected from the factors that are implicated in the increased incidence of ASD. Indeed, the hypothesis focuses on the interplay between genetic changes that could moderately affect translation in synergy with neurotoxins that elicit heat shock protein response and also affect translation, in particular through the change in the abundance of the nascent chain chaperone, heat shock 70 kDa protein 8 (HSPA8) which is required for the translation of many large or aggregation-prone proteins. We speculate that these two factors synergistically lead to protein dosage imbalance that contributes to the autistic phenotype. A recent systematic review of potential associations between environmental toxins and ASD demonstrated that the etiology of ASD is likely to involve, at least in a subset of children, complex interactions between genetic factors and environmental toxicants that might act synergistically or in parallel during critical periods of neurodevelopment [94]. The role of nonsense and missense mutations in these interactions is indeed far from being clear. There are indications that some *de novo* non-synonymous substitutions could be associated with various intellectual disabilities but the functional impact of these mutations is not well understood [45]. In general, ASD is a complex, multifactorial disorder [95-98] which makes construction of straightforward explanatory models highly problematic. Currently, we are still at the stage of searching for factors that are significantly and substantially associated with ASD.

On a more technical level, I would draw attention to the statistical testing suggested in the manuscript. “Statistical significant shift from optimal” codon frequencies may well agree with a random drift: assuming that all codons are in near-optimum (which is not true, of course) the overwhelming majority of possible random synonymous substitutions will inevitably push the distribution of codons away from optimum. I think, this particular test does not really support or refute the hypothesis. Essentially, it says that random synonymous nucleotide changes are very unlikely to result in low-entropy optimal codon distribution.

Authors’ response: The problem with models of random mutations does exist in many applications [99,100]. For example, a model proposed by McFarland and co-workers [100] accounts for three types of synonymous substitutions: transversions, CpG to TpG transitions, and all other transitions. We implemented a more complex statistical model of random mutations which takes into account the frequencies of substitutions for each nucleotide and the context of the mutating sites in the case of CpG dinucleotides (distinguishing mutations in the first and second CpG positions, see text for more detail). We believe that this model captures statistical properties of background mutation process in detail and is similar to the model developed for human germ-line mutations [100,101]. Although under this model, we showed the shift of the generated random mutations away from the optimum for AVs, the shift was substantially smaller than that observed with the actual AVs in the ASD patients (Figure 1). Notably, this was not the case for some other conditions. For example, for *de novo* mutations found in patients with schizophrenia, the opposite shift from less optimal to more optimal codons was observed (see more details in our response #3 to Dr. Smalheiser below), and in this case, random mutations under the same model also exhibit such a shift. Thus, the outcome of the model of random mutations strongly depends on the frequencies and context of the analyzed variations/mutations.

### Reviewer #2: Neil R. Smalheiser, University of Illinois at Chicago, United States of America

This article provides strong evidence that synonymous genetic variants associated with autism tend to produce less-favored codon usage, which provides further evidence in favor of the notion that impaired translation is a key risk factor, and possibly a core pathogenetic feature, of autism.

I recommend that the authors make the following clarifications:

1. Can you comment on Shank3, which has 27 synonymous variants? Is there any chance that this single case is skewing the results from the entire set? Is there anything in particular about Shank3 that makes it particularly susceptible to control via translational rate?

Authors’ response: SHANK3 is indeed an outlier in terms of the number of AVs (Table 1), one possible explanation is that this gene has a long coding sequence (5244 nucleotides). In order to test for potential skew of the results by the excessive amount of rare variations in this gene, we performed an additional experiment. We randomly sampled S% out of 27 AVs observed in SHANK3 without changing the rest of the AV set (S = 10%, 25%, 33%, 50%). This sampling was repeated 100 times for each value of S. In all 400 experiments, the P value was less than 0.01, even for S = 10% (3 AVs in SHANK3). This result indicates that the excess of mutations in SHANK3 does not significantly influence in any way conclusions of our study.

2. To extend the comment about Shank3, is there anything ELSE in terms of features of the autism gene set which would render them particularly susceptible to control via codon usage? These might be covariates that work together with codon usage and are worth knowing about, if they exist.

Authors’ response: We certainly agree that this is the key issue. We looked for such correlates of codon usage but failed to detect anything obvious. Nevertheless, we speculate that heat shock protein response (e.g., HSPA8 upregulation) is involved as an amplifier of the codon usage effect for large, aggregation-prone proteins and membrane proteins that are translocated in a semi-folded state.

3. Can you contrast the autism data with some other diseases that do NOT show the same trend? Maybe a brain disease whose incidence has not been increasing recently, and maybe a non-brain disease, etc. It would be very helpful to define neuropsychiatric disorders according to those which do, and which do not, show a relation of synonymous variants to less favored codons.

Authors’ response: This is an important question that certainly has to be addressed in the future. So far we analyzed limited sets of *de novo* mutations and synonymous variations associated with schizophrenia extracted from three previous studies [102-104]. For *de novo* synonymous mutations, we did not find any significant codon usage shift [P(S_H_ ≤ S_H__r) = 0.868 for 27 mutations in 27 genes, S_H_ = −6.12]. For the rare synonymous variations we did not find any trends either [P(S_H_ ≤ S_H__r) = 0.804 for 5 mutations in 2 genes, S_H_ = 0.98]. Thus, unlike the case of ASD described in the present paper, we did not observe any significant shift towards more rare codons although these preliminarily results should be interpreted with caution given the small sample sizes.

4. The title talks about translation “in neurons”, but is that warranted? What is the evidence that neurons are the critical compartment? All neurons? But not glial cells? Neuronal cell body vs. local translation in dendrites or at synapses? Most of the autism gene set are not brain specific, either, so it is conceivable that changes in other organs are contributory (e.g., liver, if it is detoxifying environmental toxins?).

Authors’ response: The problem of cell/tissue specificity of gene sets is indeed important. We used published gene sets from two studies [41,42] assuming that these were well curated and have biological implications in ASD. One of the theories of autism development involves excessive neuron number and atypical neural “connectivity” [105-107]. Some microglia activation was observed in autistic brains together with aberrantly close microglia-neuron associations whereas the organization of microglia itself appeared normal [108]. Although aberrant synaptic pruning was implied in pathogenesis of autism, it might be too early to address finer defects inside neurons [109]. However, recent studies have shown clear benefits of glutamate blockers in a number of animal models of ASD and fragile X syndrome [110-112]. Antagonists of metabotropic glutamate receptor subtype 5 (mGluR5) currently are in clinical trials for fragile X syndrome (~30% of patients meet the diagnostic criteria for autism) [113], a major genetic cause of intellectual disabilities. This signaling pathway is related to the regulation of synapses and neuronal circuits, and therefore neurons are the likely primary target of translational defects in autism. However, liver function is directly connected to brain health and function and recently a case study has been published in which liver transplant reversed autistic symptoms and mental delays in a child [114]. Thus, we cannot exclude a role of protein expression in liver in the etiology of ASD although there are no detoxification enzymes on our list of genes with ASD-related mutations (Table 1).

5. The least satisfying part of the paper is the discussion/speculation how translational defects might interact with environmental toxins. If the relation between synonymous changes and codon usage is truly specific to autism, that might explain why the incidence of autism is selectively increasing (when combined with environmental factors). It would help to have a better framework of exactly how the two effects might interact or synergize, than you have currently.

Authors’ response: We believe that we did our best to outline potential biological links between the shift in codon usage and putative environmental factor(s), without overstating the case which is far from being clear at this stage. We suggest that heat shock response in a high neurotoxin load environment would synergize with the codon usage effect. One example of an increasingly used toxin is sodium azide that, in addition to its well-known effects as an inhibitor of cytochrome c oxidase [90,115,116] and a stimulant of soluble guanylate cyclase [117], has been shown to inhibit translation initiation at millimolar concentrations [118]. Furthermore, micromolar exposures in cell cultures to sodium azide have been reported to cause cytoplasm to nucleus translocation of inducible forms of Hsp70 and of Hsp40, and overall upregulation of heat shock protein response [90], and accordingly, would affect translation through nascent chain chaperone function of inducible forms of Hsp70 and other heat shock proteins [81,82]. Given the apparent importance of defects in translation and in particular in translation repression [7,38], increased exposure to sodium azide might contribute to the observed increased prevalence of ASD synergistically with rare synonymous variations and mutations impairing translation, even without an increase in the rate of such variations/mutations. Sodium azide is widely used as inhibitor of bacterial growth, pesticide, and most important, propellant for car air bags with an uncertain fate in the environment [119]. All these indications notwithstanding, it should be noted that sodium azide is discussed as a potential culprit in the ASD surge as an example. Many other growing environmental hazards could be at fault alternatively or additionally.

6. Is this paper relevant to your arguments? Petrovski S, Wang Q, Heinzen EL, Allen AS, Goldstein DB. Genic intolerance to functional variation and the interpretation of personal genomes. PLoS Genet. 2013; 9(8): e1003709.

Authors’ response: This paper is indeed relevant because it emphasizes that *de novo* synonymous mutations are likely to be a good control set; we now cite this reference in the discussion of this issue.

7. In your final paragraph, you state that it is “clear” how to test your hypothesis. I think it would be better to spell out some specific tests anyway, especially focusing on the best and most critical tests.

Authors’ response: One of the venues for future research could be development of animal models to directly test our hypothesis. In our opinion, valproic acid and sodium azide are appropriate neurotoxins to study autistic behavior in mice [91]. One could analyze phenotype of already developed SHANK3 mutant mice in the presence of neurotoxins [120]. Another possibility is to construct mutated SHANK3 mice with changes in codon usage, e.g. using some of the 27AVs from our list.

### Reviewer #3: Shamil R. Sunyaev, Brigham & Women’s Hospital and Harvard Medical School, United States of America

This manuscript addresses potential molecular mechanisms underlying genetic susceptibility to autism. The authors suggest that synonymous mutations impacting translation efficiency may play a significant role. This is a highly important area of research, and the authors state an interesting hypothesis. However, I am not convinced by the provided evidence.

1) There are no data on the importance of *de novo* synonymous mutations in autism. The only convincing enrichment has been observed for nonsense and frameshift mutations, the mutations of very large effects. There is no signal for any other class of mutations. Incorporation of an unpreferred codon is unlikely to be functionally equivalent to a complete loss of function. Next, the enrichment in nonsense mutations is probably limited to cases where autism is associated with intellectual disability and is not observed in patients with high non-verbal IQ. It is, therefore, likely that cases of monogenic intellectual disability are, at least partly, responsible for the observed enrichment. As far as I know, there is no evidence that synonymous mutations cause monogenic forms of intellectual disability.

Authors’ response: We are not aware of synonymous mutations that cause monogenic forms of intellectual disability. In general, monogenetic disorders (e.g., fragile X syndrome, Rett syndrome, and neurofibromatosis) that have phenotypic overlap with autism imply impaired synaptic development and function but the fraction of monogenic forms of intellectual disability among all forms of this condition is not well defined [121,122]. Some studies even question the possibility of any substantial impact of monogenic forms of ASD [97]. ASD is known to be a complex multifactorial disease [95-98,123] although several rare single-gene disorders with a high prevalence of ASD are known [38,113]. We speculate that the impact of synonymous variations/mutations is small but sufficient to produce phenotypic effects in synergy with the increasing neurotoxin load. This hypothesis is effectively orthogonal to the existence of monogenic conditions caused by synonymous mutations.

2) It is clear that *de novo* mutations would be expected to be biased towards unpreferred codons because fixation biases due to selection or biased gene conversion reduce frequencies of unpreferred codons. The authors observed a bias in favor of unpreferred codons in synonymous mutations involved in autism and in a corresponding trend in control mutations. The proper statistical test would be to compare sets of mutations in patients and mutations in unaffected controls (better in the same genes) matched for paternal age. Comparisons with a model or segregating human SNPs are not convincing.

Authors’ response: We are not sure that *de novo* synonymous mutations are”… biased towards unpreferred codons because fixation biases due to selection or biased gene conversion reduce frequencies of unpreferred codons”. We did not observe any significant trends in our study. In fact, the bias “towards unpreferred codons” is not obvious feature of *de novo* mutations, for example, a recent analysis of large collections of *de novo* mutations in diploid yeast cells did not reveal any obvious signs of selection at the level of coding sequences as suggested by tests of selection that are based on ratios of non-synonymous/synonymous substitutions and radical/conservative non-synonymous mutations [124]. In this work we observed a trend toward unpreferred codons for rare genomic variations (the analyzed AV set) as defined by two previous studies [41,42] and compared the trend in this set with those in several control sets including *de novo* mutations in ASD patients and common SNPs (Table 2). We believe that *de novo* mutations in ASD patients (Table 2) comprise the control set closest to the set of rare synonymous variations in genes associated with ASD (the studied AV set). However, we decided to follow the suggestion of the reviewer and analyzed available sets of *de novo* synonymous mutations in unaffected individuals from two previous studies [6,7]. This set contains 103 mutations (31 mutations in CpG dinucleotides). In this set of *de novo* synonymous mutations, we did not observe any significant codon usage shift [P(S_H_ ≤ S_H__r) = 0.485]. The current data on *de novo* mutations do not allow comparisons of “sets of mutations in patients and mutations in unaffected controls (better in the same genes) matched for paternal age”, however, we find it unlikely that even much larger sets of *de novo* mutations (controlled for genes and parental age) could change the conclusions of the present work. Once again, the only trend that we observed is the significant codon usage shift for rare synonymous variations in genes associated with ASD (the AV set, Table 1). However, it is possible to use another control set for the AV set, namely rare synonymous variations that have been detected in the AV set of genes (Table 1) from unaffected siblings of ASD patients (controls according to Kelleher and co-workers [42]). Unfortunately, this set contains only 51 rare synonymous variations that is why we have not included it in the original version of this paper. Nevertheless, analysis of these rare variations revealed a trend of codon usage shift that is fully consistent with our hypothesis: the S_H_ value for the rare synonymous variations in unaffected siblings was extremely small (S_H_ = 1.4), in a sharp contrast with the AV set (S_H_ = 22.7). Moreover, the observed value S_H_ = 1.4 is significantly smaller compared to the corresponding set of random synonymous mutations [mean S_H__r = 8.2, P(S_H_ ≥ S_H__r) = 0.045]. These observations suggest that rare synonymous variations in unaffected siblings of ASD patients have negligible effect on translation of the respective mRNAs although these findings should be interpreted with caution given the small sample size.

## Abbreviations

ASD: Autistic spectrum disorders; AVs: Rare synonymous variations in genes associated with ASD; SNP: Single nucleotide polymorphism; ESEs: Exonic splicing enhancers; ESSs: Exonic splicing silencers; Hsp70: the heat shock protein 70 kDa family.

## Competing interests

The authors declare that they have no competing interests.

## Authors’ contributions

EP, EVK and IBR incepted the study. EP and IBR implemented the tests and performed data analysis. EP, EVK and IBR wrote the manuscript which was read, edited, and approved by all authors.

## Supplementary Material

Additional file 1 List of rare synonymous variations in genes associated with ASD (the AV set) used in this study. Click here for file

Additional file 2 Distribution of rare synonymous variations in genes associated with ASD (the AV set) across protein-coding sequences of the 19 genes. Click here for file
