# Supplementary material for: Impairment of translation in neurons as a putative causative factor for autism
Source: Biol Direct. 2014 Jul 10;9:16. doi: 10.1186/1745-6150-9-16 (PMC4099083; doi:10.1186/1745-6150-9-16)
Supplement: Additional file 1 — List of rare synonymous variations in genes associated with ASD (the AV set) used in this study. [file 1745-6150-9-16-S1.doc]

Additional file 1. List of AVs used in this study. Asterisks indicate that rare synonymous variants are located in CpG dinucleotides. The first three positions in the oligonucleotide correspond to codons, this context uniquely defines positions of AVs in cDNA sequences.

Gene: MAP2K1

CCC>CCT* CCCGCAATCCGGAACCAGATCATAAG

Gene: MAP2K2

GTC>GTA GTCACCAAAGTCCAGCACAGACCCTC

GAC>GAT* GACGGGGAGATCAGCATTTGCATGGA

GCG>GCA* GCGGTTCTCCGGGGCTTGGCGTACCTCCG

Gene: PIK3CA

CCC>CCG* CCCAGGTGGAATGAATGGCTGAA

GAA>GAG GAACAGGCTATGGAACTTCTGGACTGTA

ACA>ACG ACACTTCAAATTATTCGTATTATGGAAAA

ACC>ACT ACCCTAGCCTTAGATAAAACTGAGCAAGAG

Gene: UBE3A

GAC>GAT GACCCCCTGGAAACTGAACTTGGTGTTA

Gene: SHANK2

GGC>GGT* GGCGAGATCCCCCTTCACCGCGGTGAC

CCG>CCA* CCGGCGGGCTCAGACATGAACTCTGT

ACG>ACT* ACGATGCGAAGGCAGAAATCAATAGAC

GAG>GAA GAGGACAGCCCCGAGAAGACGTGCTCC

CCG>CCA* CCGAGCCAGCTGCGGCCTGACGAAAGC

GAC>GAT* GACGCCACTAAGCTGGACAACGCCCTG

CCC>CCT* CCCGGCAGAACCATCGTCGCGGTGGGC

GCC>GCT* GCCGACTCTGGGATCGAGGAGGTGGAC

Gene: FMR1

CGG>CGA CGGCAAATGTGTGCCAAAGAGGCG

Gene: SHANK3

TCG>TCA* TCGGATGTTGTACCATTCAGGG

ATC>ATT* ATCGAGGTGAACGGGGTGAAC

CCC>CCG CCCAAGAGGGCCCCCAGCACC

GAG>GAA GAGCGGCGCCGCTCCACTGTG

GCT>GCC GCTTTCTCCCCACGGAGCCCAG

AAG>AAA AAGGTCCCCCGGGAGGAGCGGA

GGC>GGT GGCTGGCAGGAGATCCACCAGGCC

CCG>CCA* CCGGCCGACTGCGTGGAGGAAGTG

GGG>GGA GGGCTCCCTGGGCCTGAAGACGAC

TCC>TCT TCCCCCTCGCCGTCGCCGCTGCCC

CCC>CCT* CCCGCGTCCGGCCCCGGCCCCGGC

AAC>AAT* AACGCCACGGACCTGCTAAAGGTG

CCG>CCA* CCGGGCTCCTTGCGGAAGGGGA

TAC>TAT* TACGGCGCGGGCGATGGCCCGG

TCC>TCT TCCCAGGCGCCCTCCCGGTCC

CCC>CCT* CCCGGCCCCGGCGCCCCCGGCC

GCC>GCT GCCCACCTGGACTTCCGCACT

AGC>AGT AGCTGTGCTCGTGTCCTGCTCTTCCG

GGG>GGT GGGGGGGCGCCCCTCCCTCCCCCTGGCG

ACG>ACT* ACGCCCCTGCCCTACCTGGAGTTTCGAT

GAG>GAA GAGAGCAACATCAGTGGCCCTTTAGCA

CCG>CCA* CCGGCCCCCATGCAGTCAGCGGCTGTGGC

CCC>CCT CCCAGCTCACCCCTGGCCCTTGCCCTGG

CCC>CCT CCCAGCCCGGCCTCAGGGAAGCCCAGC

ATC>ATT* ATCGCAGCAGCTCGGCTCTTCAGCAGCCTC

CGC>CGT* CGCGACCGCTTCGAGGACCATGAGATAGA

CCC>CCT* CCCGTGGAGAGCCGGGGGCTCCCT

Gene: AUTS2

CCA>CCG CCACAGGGCCCTCCTGAGGCCCAGCTC

Gene: CADPS2

CCC>CCT CCCATACCAGCAGAAGAGGTGAAGAAAGT

ACA>ACC ACAGAATATGCCAAAATAGAAGAGACCATG

Gene: TSC1

CCA>CCG CCACATGACAAGCACCTCTTGGACAGGA

GCC>GCT* GCCGCCTATCGGAAAGAGCTAGAAAAA

CAC>CAT CACAACGGTGAGACCAAGACCCCCAGG

Gene: TSC2

TGC>TGT* TGCGTCCGGACCGCGTCCTCTGTGGACA

CCG>CCT* CCGAAGAGGTCTCCTGTGGGCGAGTT

TCC>TCT TCCAGCTCCCCCCGCTCGCCCAGTGGCC

CTG>CTA CTGGACTACGAGTGCAACCTGGTGTCCC

GTG>GTA GTGCATCATAGCCGCTCCAACCCCACCG

Gene: NRXN1

GGC>GGA* GGCGCCGAGGGCCAATGG

GGC>GGT* GGCGACCAAGGTAAA

GCC>GCT* GCCGGAGCCAGGAACTTA

GGC>GGT* GGCGGGAAAGAGCAGGGC

CCA>CCG CCAGGCTCAGCAGAAGTGATC

Gene: NLGN3

TAC>TAT* TACGCAGCTCCCCCGATCGGC

Gene: MECP2

AGC>AGT* AGCGGCACCACGAGACCCA

AGC>AGT* AGCGTCTGCAAAGAGGAGA

AGC>AGT* AGCGGCACCACGAGACCCAAG

AGC>AGT* AGCGCCTCCTCACCCCCCAAG

GCC>GCG* GCCAAAAAGAAAGCCGTGAAGG

ACC>ACT* ACCGTACTCCCCATCAAGAAGC

AAA>AAG AAAAGCAAGGAGAGCAGCCCCA

CCC>CCT CCCCCACCTCCACCTGAGCCCG

GGG>GGT GGGAGTGTGGTGGCAGCCGCTGCC

Gene: FOXP2

GGC>GGT GGCCAGGCAGCACTTCCTGTCCAATCGCTG

Gene: RBFOX1

ACG>ACA* ACGGCCCCTCATCCCCACCCCGC

GAC>GAT* GACGCAGCACCGACGGATGGC

CGC>CGT CGCACCGTGTACAACACCTTCAGGGC

Gene: GRM1

GAC>GAT GACACTTTGCAGGCAAGGGCCATGCTTG

CGC>CGT CGCCTCTTGGTTGGCCTCTCCTCTGC

AAC>AAT AACTTCAACGAGGCCAAATATATCGCGTTC

ACC>ACT ACCCTTTACAACGTAGAGGAGGAGGAGGA

Gene: GRM5

GCT>GCC GCTCACATGCCGGGTGACATCATTATTG

ACG>ACA* ACGGTGAGAGGTCTGCTGATGGCCAT

ACA>ACG ACACATCATGTTCAGGATTCCAAAATGG

GTT>GTA GTTGCCCTCTTTATAATGGAGCCTCCTG

TTT>TTC TTTGGCAGCAACTACAAAATCATCACC

ACG>ACA* ACGTGGGCCCAGAATGAGAAGAGCAGC

AGC>AGT AGCGAGCTCAACTCCATGATGCTGTCC

Gene: HRAS

CCC>CCG CCCCCGGGACCCATGTGACCCAGCGGCCC
