# Supplementary material for: Impairment of translation in neurons as a putative causative factor for autism
Source: Biol Direct. 2014 Jul 10;9:16. doi: 10.1186/1745-6150-9-16 (PMC4099083; doi:10.1186/1745-6150-9-16)
Supplement: Additional file 2 — Distribution of rare synonymous variations in genes associated with ASD (the AV set) across protein-coding sequences of the 19 genes. [file 1745-6150-9-16-S2.doc]

Additional file 2. Distribution of rare synonymous variations in genes associated with ASD (the AV set) across protein-coding sequences of the 19 genes (Table 1). (A) Number of AVs in the intervals 1-100 nucleotides (nt), 101-200 nucleotides, 201-300 nucleotides, 301-400 nucleotides, 401-500 nucleotides. (B) Number of AVs in the intervals 1-10%, 11-21%, …, 91-100%, the length of each coding sequence was taken as 100%. The numbers of mutations in the intervals 1%-50% and 51%-100% are 36 and 51 (P = 0.067, no significant difference, the sign test).

(A)

(B)
